# Assessment of ICU nurses’ competency towards delirium among critically ill patients

**DOI:** 10.1186/s12912-024-02330-z

**Published:** 2024-10-19

**Authors:** Mona Ibrahim Hebeshy, Samia Hussieny Gaballah, Noha Mohammed Ibrahim

**Affiliations:** 1 Medical-Surgical Nursing, Faculty of Nursing, Suez Canal University, 4.5 KM the Ring Road, Ismailia, 41522 Egypt; 2 Department of Nursing, College of Applied Medical Sciences, University of Bisha, P.O Box- 551, Bisha, Saudi Arabia; 3 Medical-Surgical Nursing, Faculty of Nursing, Port Said University, Port Said, Egypt

**Keywords:** ICU nurses, Delirium, Knowledge, Practice, Attitudes, Patient outcomes

## Abstract

**Background:**

ICU nurses play a critical role in managing delirium in critically ill patients, yet their competency in this area remains under-explored.

**Aim:**

To assess ICU nurses’ competency including their knowledge, practice, and attitudes toward delirium among critically ill patients.

**Methods:**

A correlational descriptive study was conducted with 67 nurses in the medical intensive care unit at Suez Canal University Hospitals. Data was collected using an online survey and analyzed using descriptive statistics, ANOVA, and Pearson’s correlation.

**Results:**

Nurses showed a moderate knowledge level (M = 8.55), a low practice level (M = 6.62), and positive attitudes (M = 21.65) toward ICU delirium. ANOVA results indicated significant differences in practice scores based on educational level, *F* (2, 64) = 3.361, *p* = .041, and in knowledge scores based on ICU experience, *F*(3, 63) = 6.455, *p* < .001. Nurses with Master of Science in Nursing (MSN) degrees had higher practice scores than those with diplomas or Bachelor of Science in Nursing (BSN) degrees. There were no significant differences in knowledge and attitude scores based on educational level or age. Gender differences were minimal, with male nurses slightly outperforming female nurses. Correlation analysis showed positive relationships between age, education, ICU experience, and ICU nurses’ levels of knowledge and practice.

**Conclusion:**

ICU nurses demonstrate a knowledge-practice gap in delirium management. Targeted educational interventions, mentorship programs, and a focus on practical skills are essential to enhance delirium care.

**Relevance to clinical practice:**

These findings highlight the urgent need for comprehensive delirium education and training programs for ICU nurses. By improving nurses’ knowledge and practice, healthcare organizations can significantly enhance the early detection, prevention, and management of delirium, ultimately leading to improved patient outcomes and reduced length of stay in the ICU.

## Background

Delirium remains a prevalent and concerning issue in intensive care units (ICUs), affecting a significant proportion of critically ill patients. It is characterized by acute attention, awareness, and cognition disturbances, often fluctuating in severity throughout the day [1, 2] About 2 out of 3 patients in the ICU develop delirium, and more than 7 million hospitalized Americans suffer from delirium each year [3]. Typically, delirium arises in approximately 20–50% of patients who are critically ill and not receiving mechanical ventilation, and in 50–80% of patients who are mechanically ventilated [4–7].

Common risk factors for ICU delirium include environmental factors such as excessive light and noise, disturbance of the sleep-wake cycle, repetitive interventions by healthcare professionals, severity of illness, and the use of sedatives and multiple drugs. Additional contributing factors include age, emotional characteristics, sepsis, and hypoxia [8, 9]. Delirium is more prevalent in older adults due to their increased vulnerability to contributing factors, including preexisting cognitive impairments and polypharmacy [8].

The reported incidence of delirium can vary widely based on the characteristics of the population being studied, such as age, medical condition, and severity of illness, as well as the diagnostic and screening tools used. Critically ill patients, particularly older adults, are at increased risk for developing delirium. These patients are more likely to experience a cascade of negative outcomes, including prolonged ICU stays, increased risk of rehospitalization, and higher mortality rates [10, 11]. Additionally, delirium has been associated with prolonged mechanical ventilation, increased likelihood of discharge to long-term care facilities, and substantial healthcare costs. Moreover, cognitive impairments resulting from delirium, such as memory loss, difficulty concentrating, and problems with reasoning, can persist for months or even years post-discharge [4, 12, 13].

In this context, the competency of ICU nurses plays a pivotal role in the early identification and management of delirium. Benner defined competence as the ability to perform nursing tasks by integrating knowledge to achieve desirable outcomes [14]. In the ICU, nursing competence involves the effective integration of knowledge, attitude, and skills into patient care, ensuring high-quality, ethical, and safe nursing practice [15]. Given their close proximity to critically ill patients, ICU nurses are in a unique position to prevent and screen for delirium early. However, lacking essential knowledge and skills may prevent timely intervention, potentially leading to adverse outcomes.

Al-Dossary et al. noted that nurses with sufficient knowledge reserves are better equipped to adopt coping strategies as patients’ conditions evolve [17]. Nurses with a positive attitude toward delirium management are more likely to engage actively in clinical practice. Therefore, understanding nurses’ knowledge, attitudes, and practices (KAP) toward delirium can identify gaps in their evaluation practices and form the basis for appropriate preventive measures.

Despite the established importance of ICU nurses in delirium management, gaps remain in understanding the extent of their competency in this area. Previous research has not fully explored how ICU nurses’ knowledge, attitudes, and practices influence the early detection and intervention of delirium. Additionally, there is limited information on how these factors vary across different ICU settings and among nurses with varying levels of experience. This study aims to assess the competency of ICU nurses toward delirium among critically ill patients, shedding light on knowledge, attitudes, and practices that may influence the early detection and intervention of this complex condition.

### Aim of the study

This study aimed to assess the competency of ICU nurses toward delirium among critically ill patients, with a specific focus on examining the relationship between nurses’ knowledge, practices, and attitudes toward delirium and their sociodemographic characteristics.

### Research questions

1. What is the level of ICU nurses’ knowledge and practice of delirium?
2. What is the level of ICU nurses’ attitude toward delirium?
3. Is there a relationship between ICU nurses’ knowledge, practice, and attitudes toward delirium among critically ill patients and their sociodemographic characteristics?

## Method

### Research design

This study employed a cross-sectional descriptive correlational design to assess the competency of ICU nurses toward delirium among critically ill patients. This design is a commonly used method in nursing research to explore complex phenomena and describe relationships among variables without inferring causality. A cross-sectional design was chosen for its efficiency in collecting data at a single point in time. This approach allowed for a comprehensive assessment of ICU nurses’ knowledge, attitudes, and practices toward delirium, as well as their relationship with sociodemographic characteristics.

### Setting

The study was conducted in the medical intensive care unit (ICU) at Suez Canal University Hospital between September 2023 and March 2024. The hospital serves a diverse population and functions as a prominent center for healthcare education, training, and research in the Suez Canal region. The ICU has 16 beds and admits critically ill adult patients with medical and surgical conditions. The unit is staffed by full-time registered nurses who work in three rotation-based shifts per day, providing a nurse-to-patient ratio of 1:1 or 1:2.

### Sampling

A convenience, nonprobability sample was used, comprising staff nurses who meet the inclusion criteria and volunteer to participate in this study. The inclusion criteria were: (a) nurses who provide direct clinical care and conduct evaluations and documentation for critically ill patients in the ICU; (b) nurses with more than six months of ICU nursing experience; and (c) male and female nurses.

### Sample size calculation

A prior power analysis was conducted to determine the adequate sample size for the statistical analyses used in this study. The analysis considered an effect size of 0.40, a significance level (alpha) of 0.05, and a desired power of 0.80. The results of the power analysis indicated that a sample size of 67 participants was sufficient to achieve adequate statistical power for both the ANOVA and correlation analyses performed.

### Measurements

For data collection, the measurement tools used in this study included ICU Delirium Knowledge and Practice Questionnaire and ICU Delirium Attitude Scale.

### ICU delirium knowledge and practice questionnaire

To assess nurses’ knowledge and practice related to delirium, researchers developed the ICU Delirium Knowledge and Practice Questionnaire. This tool was created based on previous research [18, 19] and reviewed by experts in intensive care for clarity, relevance, and content validity. The questionnaire consisted of two parts. The first gathered basic information about the nurses, such as their age, level of education, and years of ICU experience. The second part contained questions to evaluate nurses’ knowledge and practices regarding delirium. Nurses answered “yes” or “no” to each question. A correct answer was scored as “1,” and an incorrect answer as “0.” A total knowledge score was calculated based on the first 11 questions, ranging from 0 to 11. Similarly, a practice score was determined from questions 12 to 20, with a possible range of 0 to 9. To ensure the reliability and validity of the questionnaire, a rigorous validation process was implemented. Content validity was confirmed through expert review, in which subject matter experts evaluated the relevance and comprehensiveness of the items in covering the key aspects of delirium management. Reliability was tested in a pilot study involving ICU nurses, with internal consistency measured by Cronbach’s alpha. The resulting Cronbach’s alpha coefficient of 0.75 indicates an acceptable level of reliability.

### ICU delirium attitude scale

The Delirium Attitude Scale, developed by Devlin and colleagues in 2008, was used for assessing healthcare professionals’ attitudes toward patients experiencing delirium [20]. It consisted of 8 items, each rated on a 5-point Likert scale ranging from “strongly agree” to “strongly disagree.” Higher scores on the scale indicated a more positive attitude, while lower scores reflected a more negative attitude toward delirium.

### Data collection

Prior to data collection, the ICU Delirium Knowledge, Practice, and Attitudes Questionnaires pilot tested in a convenience sample of 10 intensive care nurses to assess face validity and clarity. Minor revisions were implemented based on the pilot data, and nurses who participated in the pilot testing were excluded from the actual study to maintain data integrity. Participants were contacted personally and informed about the study’s goals—those who agreed to participate provided informed consent and completed the questionnaire online. Questionnaires were administered electronically, with an average completion time of 5–10 min.

### Ethical considerations

The Research Ethics Committee, Faculty of Nursing, Suez Canal University, Egypt, approved the current study. Permission to collect data was obtained from the hospital and ICU administrative authorities. The third author obtained informed consent from all participants after explaining the study’s purpose and the significance of the current research. Nurses were also informed that they had the right to refuse participation or withdraw from the study whenever they wanted to without any harm. Anonymity and confidentiality were ensured through the use of coded data, with each participant assigned a unique identifier to separate personal information from the responses. To secure confidentiality, the use of encrypted survey was used. All data were stored in password-protected files accessible only to the research team. Moreover, nurses were informed that the collected data would not be reused in future studies without their explicit permission. Moreover, nurses were informed that these data would not be reused in another study without their permission. Written informed consent was obtained from the nurses who agreed to participate.

### Data analysis

Data were analyzed using (SPSS) software package. First, the compatibility of all data to normal distribution was examined. The demographic characteristics of the nurses were described using means and percentages. The levels of nurses’ knowledge, practice, and attitudes toward delirium were assessed using mean and standard deviation.

Given that the data met the assumptions of normality, parametric tests were selected for further analysis. A t-test was used to evaluate differences in knowledge, practice, and attitude scores between two groups of nurses, while one-way ANOVA with Post-hoc Tukey Honestly Significant Difference (HSD) was employed to assess differences across multiple groups based on sociodemographic characteristics (e.g., age, education, years of experience). These tests were chosen because they are suitable for comparing means between groups when the data is normally distributed, and the sample sizes are adequate.

Pearson product-moment correlation analysis was performed to explore the relationships between nurses’ knowledge, practice, attitudes, and their sociodemographic characteristics. Pearson’s correlation was selected due to its ability to measure the strength and direction of linear relationships between continuous variables. A *p*-value of ≤ 0.05 was considered statistically significant.

## Results

### Characteristics of nurses

The study included 67 nurses working in ICU at Suez Canal University Hospitals. The majority of the participants were females (76.1%) and aged 18–25 years. Of the nurses, 50.7% had at least a bachelor’s degree. The majority of the nurses were on the job for less than one year of experience working in intensive care (38.8%) (Table 1).

**Table 1:** Distribution of the nurses according to their descriptive characteristics (*n* = 67)

Descriptive Characteristics		*N*	%
Age	20	1	1.5
	18–25	34	50.7
	26–35	26	38.8
	36–45	4	6.0
	> 45	2	3
Gender	Male	16	23.9
	Female	51	76.1
Level of education	Diploma	27	40.3
	BSN	34	50.7
	MSN	6	9.0
Years of ICU experiences	< 1	26	38.8
	1–3	22	32.8
	4–6	13	19.4
	> 7	6	9

### Nurses’ knowledge, practice, and attitudes on ICU delirium

The average scores for ICU nurses regarding delirium were 8.55 (*SD* = 1.90) [range: 2 to 11] for knowledge, 6.62 (*SD* = 1.67) [range: 2 to 9] for practice, and 21.65 (*SD* = 4.71) [range: 7 to 35] for attitude.

### Differences in ICU nurses’ knowledge, practice, and attitudes toward delirium based on the level of education

The analysis of variance (ANOVA) revealed significant differences among ICU nurses’ practice and their levels of education, *F* (2, 64) = 3.361, *p* = .041. Post hoc analyses using Tukey’s Honestly Significant Difference (HSD) test indicated that participants with a Master of Science in Nursing (MSN) degree had significantly higher scores (M = 1.85) than those with a Diploma (M = − 0.116, *p* = .036) or a Bachelor of Science in Nursing (BSN) degree (M = -1.73, *p* = .048) on the practice. However, there were no significant differences between participants with a Diploma and those with a BSN degree (*p* = .958).

No significant differences were found among the levels of education for ICU nurses’ knowledge, *F* (2, 64) = 0.552, *p* = .579, and attitude, *F* (2, 64) = 1.510, *p* = .229. Additionally, effect size estimates indicated small effects for knowledge (η² = 0.017), moderate effects for practice (η² = 0.095), and small effects for attitude (η² = 0.045) (See Table 2).

**Table 2:** Educational level differences in nurse knowledge, practice, and attitude toward ICU delirium (*n* = 67)

		Sum of Squares	Mean Square	Eta squared (η²)	F	Sig.
Knowledge	Between groups	4.045	2.023	0.017	0.552	0.579
	Within groups	234.522	3.664			
Practice	Between groups	17.647	8.823	0.095	3.361	0.041*
	Within groups	168.025	2.625			
Attitude	Between groups	66.089	33.045	0.045	1.510	0.229
	Within Groups	1401.015	21.891			

*p < .05

### ICU years of experience differences in nurse knowledge, practice, attitude toward ICU delirium

The ANOVA results indicated a significant difference among levels of ICU years of experience and ICU nurses’ knowledge, *F* (3, 63) = 6.455, *p* < .001. Post hoc Tukey’s HSD tests revealed that participants with less than one year of ICU experience had significantly lower scores on knowledge compared to those with 1–3 years (Mean Difference = -1.982, *p* < .001), 4–6 years (Mean Difference = -1.346, *p* = .102), and more than 7 years (Mean Difference = 0.153, *p* = .997) of experience. Moreover, nurses with 1–3 years of experience exhibited significantly higher scores compared to those with less than one year (Mean Difference = 1.982, *p* < .001) and more than 7 years of experience (Mean Difference = 2.136, *p* = .040). No significant differences were found between nurses with 4–6 years and those with less than one year (*p* = .102) or more than 7 years of ICU experience (*p* = .290) in their knowledge.

For the ICU nurses’ practice, no significant difference was found among levels of ICU years of experience, *F* (3, 63) = 1.730, *p* = .170. Similarly, there was no significant difference between levels of ICU years of experience and ICU nurses’ attitude, *F* (3, 63) = 0.116, *p* = .950. Effect size estimates showed a large effect for knowledge (η² = 0.235), a small effect for practice (η² = 0.076), and a very small effect for attitude (η² = 0.005). In summary, the ANOVA results indicate significant differences in knowledge among ICU nurses with different levels of experience, but no significant differences in practice and attitude (Table 3).

**Table 3:** ICU years of experience differences in nurse knowledge, practice, and attitude toward delirium (*n* = 67)

		Sum of Squares	Mean Square	Eta squared (η²)	F	Sig.
Knowledge	Between groups	56.092	18.697	0.235	6.455	< 0.001^**^
	Within groups	182.476	2.896			
Practice	Between groups	14.129	4.710	0.076	1.730	0.170
	Within groups	171.543	2.723			
Attitude	Between groups	8.068	2.689	0.005	0.116	0.950
	Within Groups	1459.036	23.159			

** *P* < .001

### Age differences in ICU nurses’ knowledge, practice, and attitudes toward delirium

The ANOVA results revealed no significant differences in knowledge *F* (3, 63) = 1.290, *p* = .286, practice *F* (3, 63) = 0.358, *p* = .783, and attitude *F* (3, 63) = 0.885, *p* = .454 scores across different age categories. Effect size estimates indicated small effects for knowledge (η² = 0.058), practice (η² = 0.017), and attitude (η² = 0.040) (Table 4).

**Table 4:** Age differences in ICU nurses’ knowledge, practice, and attitudes toward delirium

		Sum of Squares	Mean Square	Eta squared (η²)	F	Sig.
Knowledge	Between groups	13.808	4.603	0.058	1.290	0.286
	Within groups	224.759	3.568			
Practice	Between groups	3.114	1.038	0.017	0.358	0.783
	Within groups	182.557	2.898			
Attitude	Between groups	59.345	19.782	0.040	0.885	0.454
	Within Groups	1407.759	22.345			

### Gender differences in nurse knowledge, practice, and attitude

Descriptive statistics indicated gender differences in nurse knowledge, practice, and attitude toward ICU delirium. Male nurses demonstrated marginally higher mean scores in both knowledge (M = 8.88) and practice (M = 6.69) compared to female nurses (knowledge: M = 8.45; practice: M = 6.61). Notably, male nurses reported significantly higher mean scores in attitude towards ICU delirium (M = 23.25) than female nurses (M = 21.16).

Independent-sample t-tests confirmed a significant gender effect on attitude toward ICU delirium, *t*(39.73) = 1.96, *p* = .028. Male nurses reported significantly higher attitude scores than female nurses. The 95% confidence interval for the difference in means was (-0.065, 4.251). No significant gender differences were found in knowledge or practice (*p* > .05) (See Table 5).

**Table 5:** Gender differences in nurse knowledge, practice, and attitude

	Levene’s Test for Equality of Variances		t-test for Equality of Means	
	F	Sig.	t	Mean differences
knowledge	7.95	0.376	0.776	0.424
Practice	0.080	0.779	0.165	0.080
Attitude	1.234	0.271	0.157	2.09

### Relationships between nurse knowledge, practice, attitude, and sociodemographic characteristics within ICU delirium care

The correlation table presents significant associations between nurse knowledge, practice, attitude, and sociodemographic characteristics in ICU delirium care.

Age exhibited positive correlations with level of education (*r* = .366, *p* < .01) and ICU years of experience (*r* = .379, *p* < .01), indicating that older nurses tend to possess higher educational qualifications and more extensive experience in ICU settings. Gender did not correlate significantly with nurse attributes, except for a negative association with attitude (*r* = − .191, *p* > .05) among female nurses, suggesting a potential gender-based difference in attitude towards ICU delirium care.

Level of education demonstrated positive correlations with nurse knowledge (*r* = .353, *p* < .01) and practice (*r* = .412, *p* < .01), indicating that nurses with advanced educational backgrounds tend to possess greater knowledge and engage in better practices related to ICU delirium management. ICU years of experience positively correlated with age (*r* = .379, *p* < .01) and total practice (*r* = .338, *p* < .01), suggesting that nurses with more ICU experience, often older individuals, tend to exhibit enhanced clinical proficiency and adherence to best practices in managing ICU delirium.

Total practice showed a strong positive correlation with total knowledge (*r* = .747, *p* < .01), emphasizing the importance of practical application in reinforcing theoretical understanding of ICU delirium care. However, total attitude did not exhibit significant correlations with any sociodemographic characteristic or nurse attribute (Table 6).

**Table 6:** Correlations between nurse knowledge, practice, attitude, and their sociodemographic characteristics

	Age	Gender	Level of Education	ICU years of Experience	Practice	knowledge	Attitude
Age	--------						
Gender	− 0.118	--------					
Level of Education	0.366**	− 0.057	---------				
ICU years of experience	0.379**	0.064	0.286*	--------			
Practice	0.315**	0.012	0.412**	0.338**	---------		
knowledge	0.203	0.069	0.353**	0.253*	0.747**	--------	
Attitude	0.066	− 0.191	0.207	0.061	0.035	0.086	--------

**. Correlation is significant at the 0.01 level (2-tailed)

*. Correlation is significant at the 0.05 level (2-tailed)

These findings emphasize the complex relationship between nurse sociodemographic characteristics and their knowledge, practice, and attitudes toward ICU delirium care, providing insights for enhancing care standards and addressing potential areas for further training and development in ICU settings.

## Discussion

The results of this study revealed a moderate level of knowledge. These results are consistent with previous research by Mathew, Ashok, and Punnoose (2024), who also identified a moderate level of delirium knowledge among nurses [20]. Several factors may contribute to this moderate knowledge level. One significant factor could be the lack of standardized education or formal training programs specifically focused on delirium management within ICU settings. Many nursing curricula may not adequately cover delirium, leading to gaps in knowledge that persist in professional practice. Furthermore, the variability in educational backgrounds, as evidenced by the differences in knowledge scores based on ICU experience, suggests that nurses with less advanced degrees or less experience may not have been exposed to the same depth of information regarding delirium as those with more advanced education or longer ICU experience.

Nurses demonstrated a low level of delirium management practice, with a mean score of 6.62, indicating a significant gap between knowledge and application. This finding is consistent with previous research by Papaioannou et al. (2023) [21]. The low practice level of nurses in delirium management can be explained by factors such as limited hands-on training opportunities, the demanding ICU environment, and the absence of clear delirium care protocols that hinder the implementation of evidence-based interventions. Furthermore, variations in nurses’ educational backgrounds and clinical experience may contribute to difficulties in applying evidence-based interventions consistently.

In contrast to previous research reporting negative nurse attitudes towards patients with delirium [16, 21, 22], our study found a positive nurse attitude, with a mean score of 21.65. While the reasons for this discrepancy require further exploration, potential factors include differences in workload and patient acuity between male and female nurses. It is possible that male nurses, often exposed to higher acuity patients, may develop different perspectives on delirium care.

Nurses with BSN or MSN degrees exhibited significantly higher levels of delirium knowledge and practice compared to colleagues with lower qualifications. These findings align with previous research highlighting the importance of advanced education in critical care [23, 24]. However, a surprising finding emerged: MSN-educated nurses demonstrated similar practice scores but lower knowledge scores than their BSN-prepared counterparts suggesting a potential disconnect between theoretical knowledge and practical application. This discrepancy may be attributed to several responses: MSN-educated nurses often undertake advanced clinical roles that require practical application of knowledge, which may enhance their practical skills in managing delirium despite not having higher theoretical knowledge scores. This practical experience could stem from more intensive hands-on training or greater exposure to complex clinical situations, leading to improved practice scores.

However, the lower knowledge scores observed in MSN-educated nurses suggest that their advanced education may not always translate into a superior theoretical understanding of delirium, possibly due to a focus on practical skills over theoretical content in their training. This discrepancy might indicate a need for educational interventions that better integrate theoretical knowledge with practical application.

Furthermore, ICU experience was crucial in influencing ICU nurses’ knowledge and practice towards delirium. With less than one year of experience, novice nurses had lower knowledge and practice scores compared to their more experienced counterparts, aligning with previous research on limited delirium knowledge among novice nurses (Papaioannou et al., 2022) [21]. This can be explained by the fact that novice nurses, with less than one year of ICU experience, are still in the early stages of their professional development and may not yet have been fully exposed to the complexities of delirium management. Their limited clinical experience can result in less familiarity with the signs, symptoms, and evidence-based interventions for delirium. Additionally, novice nurses may have had fewer opportunities to engage in advanced training or mentorship programs that could enhance their knowledge and practice in this area. The steep learning curve in the ICU, combined with the overwhelming nature of critical care, may also contribute to their lower scores.

Male nurses demonstrated slightly higher mean scores than female nurses in delirium knowledge, practice, and attitude. While differences in knowledge and practice were minimal, the gender gap in attitude was more pronounced. These findings align with previous research indicating that gender influences perceptions of delirium care [25]. Potential factors contributing to the observed gender differences include educational background, training, and confidence. The larger attitude disparity likely reflects broader societal and cultural influences on gender roles and care priorities. Despite these variations, both genders demonstrated comparable capabilities in delirium management.

Additionally, the study indicated differences in the ICU nurses’ practice among various age groups. Older nurses demonstrated higher levels of practice in delirium management compared to their younger counterparts. This disparity is likely due to the extensive clinical experience that older nurses possess, including greater exposure to delirium cases. Such experience allows for the development of refined clinical judgment and a deeper understanding of delirium’s complex manifestations. Research by Papaioannou et al. (2022) also emphasized that older nurses were more proficient at implementing evidence-based interventions for delirium management [21]. Their accumulated knowledge and skills enable them to provide more effective and timely care for patients with delirium.

The study found no significant differences in nurses’ knowledge or attitudes toward delirium management based on their educational level or age. This contradicts the common expectation that higher education and greater experience would lead to better outcomes.

Several factors may explain these findings. First, standardized delirium management curricula across nursing programs may ensure that nurses from different educational backgrounds acquire similar knowledge and attitudes. For example, the American Association of Critical Care Nurses (AACN) has developed comprehensive guidelines for delirium prevention and management, which are often incorporated into nursing curricula [26]. Second, practical experience in the ICU may be more influential than age or education in shaping nurses’ knowledge and attitudes. Studies have shown that clinical experience, rather than formal education, is often a stronger predictor of clinical competence [27]. Finally, the clinical environment and organizational culture within the ICU may play a significant role in shaping nurses’ attitudes toward delirium management. Organizational protocols, interdisciplinary teamwork, and shared experiences in the ICU may contribute to a convergence of attitudes among nurses of varying ages and educational backgrounds [28].

Correlation analyses demonstrated positive associations between nurses’ delirium knowledge and practice with both years of experience and educational levels. These findings underscore the importance of continuous learning and professional development in optimizing patient care and enhancing delirium management capabilities among ICU nurses. Previous research supports these results, with Papaioannou et al. (2022) identifying a significant relationship between education level and nurse knowledge [21]. Similarly, Hoch et al. (2022) found that experienced nurses excelled in clinical decision-making and delirium prevention [29].

Interestingly, a negative association emerged between gender and attitude towards delirium, with female nurses reporting lower scores. This finding contrasts with previous research suggesting otherwise [30, 31]. The increased stress, challenges, and workload associated with delirium management may contribute to this negative attitude among female nurses. Understanding the underlying factors influencing this gender-based difference is essential for developing targeted interventions to improve overall job satisfaction and patient care.

Finally, the correlation analyses demonstrated a positive association between age, educational level, and ICU years of experience, indicating that professional development and clinical exposure cumulatively enhance nurses’ delirium management competencies. Older nurses typically possessed higher educational qualifications and more extensive ICU experience, contributing to their advanced knowledge and practice. Notably, a strong correlation was found between ICU nurse practice and knowledge, highlighting the interdependence of theoretical understanding and practical application in delirium care. This underscores the necessity of integrating hands-on training, simulation-based learning, and continuous quality improvement into nursing education and practice to optimize patient outcomes.

## Conclusion

This study identified a significant knowledge-practice gap in delirium management among ICU nurses. Addressing this gap requires a thorough examination of systemic and organizational factors that may obstruct the application of evidence-based practices. Barriers such as insufficient resources, outdated policies, inadequate training, and cultural or contextual challenges must be addressed to enable the effective translation of knowledge into practice.

The findings highlight the critical role of nurses in managing delirium, with gender disparities observed in knowledge, practice, and attitudes. These disparities emphasize the need for targeted educational interventions that cater to the unique needs of both male and female nurses. Through comprehensive education, mentorship, and interdisciplinary collaboration, healthcare organizations can bridge the knowledge-practice gap and elevate the standard of delirium care. Implementing evidence-based guidelines and fostering a culture of continuous quality improvement are vital steps toward optimizing patient outcomes, minimizing delirium-related complications, and enhancing overall care in ICU settings.

### Implications for clinical practice

The study’s findings emphasize the critical need for improved delirium management strategies within ICU settings. The knowledge-practice gap identified highlights the urgency for targeted, evidence-based educational interventions. Comprehensive training programs that address both theoretical understanding and practical skills in delirium care can enhance nurses’ competency, leading to better patient outcomes.

One example of a successful training initiative is the ABCDEF bundle (Awakening and Breathing Coordination, Delirium Monitoring/Management, Early Mobility, and Family Engagement), widely recognized for improving delirium management in ICUs. Implementing such evidence-based protocols can bridge the gap between knowledge and practice by promoting a multidisciplinary, holistic approach to delirium care.

Mentorship programs are also crucial for fostering continuous learning and practical application. The Clinical Nurse Leader (CNL) model, for instance, pairs experienced nurses with novices, promoting skill development and evidence-based practice. Adapting this model to focus on delirium care would help ensure that theoretical knowledge is effectively translated into clinical practice.

The significant findings from the ANOVA analysis suggest that advanced education, such as an MSN degree, is associated with better practical skills in managing delirium. This indicates a need for educational curricula that blend advanced theoretical concepts with hands-on clinical training. Continuing education through workshops and seminars can help bridge any remaining gaps in knowledge, while clinical reflection and feedback opportunities can further enhance the application of delirium care principles.

Finally, interdisciplinary collaboration should be promoted to improve communication and coordination in delirium care. By integrating evidence-based delirium care pathways into routine practice and regularly evaluating their effectiveness through quality improvement initiatives, healthcare organizations can reduce delirium-related complications and improve patient outcomes. Supporting ICU nurses through education, mentorship, and collaboration is key to elevating the standard of care for critically ill patients.

### Limitations

Our study, while informative, has limitations such as a small sample size and focus on a specific geographical context (Egypt) may limit the generalizability of the findings to other regions or healthcare systems. Future research involving larger, more diverse samples is essential to validate these results. Additionally, the use of convenience sampling, chosen for its practicality and ease of access, introduces the risk of selection bias. Participants who volunteered for the study may differ from the broader population of ICU nurses in terms of motivation, experience, or interest in delirium management, potentially biasing findings. Consequently, the study’s outcomes may not fully represent the wider ICU nurse population. Future research might consider more randomized sampling methods to enhance the representativeness of the sample.

### Future research directions

Future research should address several areas to build on the findings of this study and further advance delirium management in ICU settings.

1. ***Evaluate the effectiveness of targeted delirium education programs***: Research should assess the long-term impact of these programs on nurses’ knowledge, practice, and attitudes.
2. ***Conduct longitudinal studies on knowledge application***: Longitudinal studies can provide insights into the retention and application of delirium management knowledge and skills over time, identifying areas requiring ongoing support or refresher courses.
3. ***Explore systemic and organizational barriers***: Research should examine factors such as resource limitations, policy constraints, and cultural attitudes within healthcare settings that hinder the implementation of evidence-based delirium care practices.

By addressing these areas, future research can provide a deeper understanding of how to improve delirium management and contribute to more effective, evidence-based practices in ICU settings.

## Data Availability

The data that support the findings of this study are available from the corresponding author upon reasonable request.
